# Low Radiologic Sensitivity in Detecting Radiation-Associated Breast Angiosarcoma (RAS)

**DOI:** 10.7759/cureus.36508

**Published:** 2023-03-22

**Authors:** Jonathan Fakhry, Mariam Hanna

**Affiliations:** 1 Breast Imaging, University of Florida College of Medicine, Gainesville, USA

**Keywords:** breast cancer screening, radiation therapy, lumpectomy, invasive ductal carcinoma, secondary breast angiosarcoma

## Abstract

Due to its rarity, literature pertaining to radiation-associated breast angiosarcoma (RAS) remains sparse, with most studies focusing on retrospective review. Of more significant concern is the ambiguity of screening recommendations and modalities used to detect RAS, with current guidelines focusing on yearly mammographic imaging for women who underwent lumpectomy with radiation. Unfortunately, routine post-cancer screening has demonstrated low sensitivity in detecting RAS, often mistaking it for benign changes in roughly half of cases.

We present an 83-year-old woman initially diagnosed with stage 1 invasive ductal carcinoma of the left breast who underwent a lumpectomy followed by radiation with 6040 cGy. Five years after her initial diagnosis, the patient noticed a suspicious lesion which then led her to undergo multiple modalities of imaging that described benign features. After continued concern, a biopsy was taken that demonstrated RAS of the left breast within the irradiated site. The patient underwent further radiation and declined surgical intervention.

Routine screening with mammography and ultrasonography following breast radiation treatment are not sensitive modalities in detecting RAS. High-risk patient groups treated with greater than 0.5 Gy of radiation with concerning physical features 2-10 years after treatment should undergo MRI with biopsy at the initial concern to rule out angiosarcoma. Benign findings on imaging with patients in these groups should also consider biopsy.

## Introduction

Radiation-associated breast angiosarcoma (RAS) is a rare yet feared complication of breast cancer treatment, with a reported prevalence of 0.05% [1]. RAS most often presents in older women (median age of 67-71) following an average latency of 7.3 years after radiotherapy [2]. Factors associated with an increased risk of developing RAS include prior lumpectomy, white race, radiation, and having a left-sided tumor [3]. The increasing use of radiation for breast cancer treatment, as well as earlier screening and detection, have led to concern about a potential increase in the incidence of RAS within the upcoming years [4]. Due to the presumption of scarring and prior surgery/radiation, it is not uncommon for angiosarcoma to be missed initially, even by trained radiologists [2, 5]. Further mammographic and ultrasonographic studies have also been shown to misdiagnose RAS as benign in 50% of cases, with MRI being a more reliable modality in detecting changes related to angiosarcoma [2, 6].

Here we describe a case of a woman who, after undergoing radiation therapy for invasive ductal carcinoma, developed RAS which was initially misdiagnosed as benign skin thickening associated with prior treatment on multiple imaging modalities.

## Case presentation

An 83-year-old woman arrived at the clinic for evaluation of skin alterations in her left breast that she had noticed two months prior. The patient reported periareolar skin thickening with purple raised lesions on her left breast associated with mild pain; otherwise, she was asymptomatic. Five years prior to the appointment she was diagnosed with stage I infiltrating ductal carcinoma of the left breast that was ER/PR positive and HER-2 negative. She was treated with lumpectomy with adjuvant radiation therapy. She received 5040 cGy in 28 fractions of 180 cGy/fx, one fx/day using tangents, 6 and 15 MV photons. They were followed by a boost to the lumpectomy site for an additional 1000 cGy in five fractions of 200 cGy/fx using reduced tangents, 6 and 15 MV photons. The treatment interval was one month long. The patient also complained of sharp shooting pains across her left breast that have remained constant since the lumpectomy. After her visit, an assessment was made that the skin changes were consistent with healing from past treatment, and they would not biopsy the lesion due to concerns of poor wound healing.

**Figure 1:**
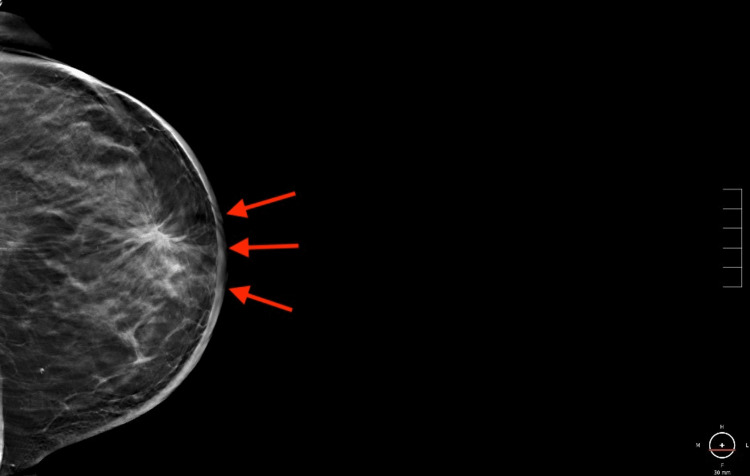
Mammogram of the left breast. Full paddle craniocaudal tomosynthesis view of the left breast demonstrating an area of distortion in the subareolar region with diffuse skin thickening. Red arrows point to the site of the lesion.

**Figure 2:**
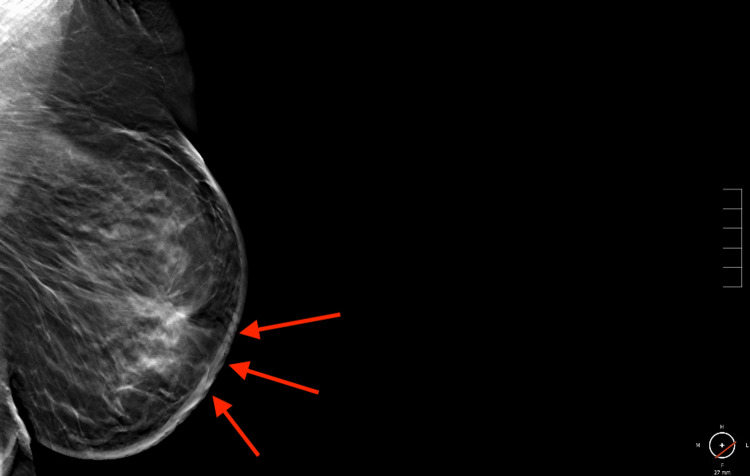
Mammogram of the left breast. Full paddle mediolateral oblique tomosynthesis view of the left breast demonstrating an area of distortion in the subareolar region with diffuse left breast skin thickening. Red arrows point to the site of the lesion.

Seven months before her initial presentation to the clinic, a repeat ultrasound with mammography (Figures 1-2) was not concerning for malignancy. Comparison with prior ultrasounds at the time did not demonstrate significant alterations.

**Figure 3:**
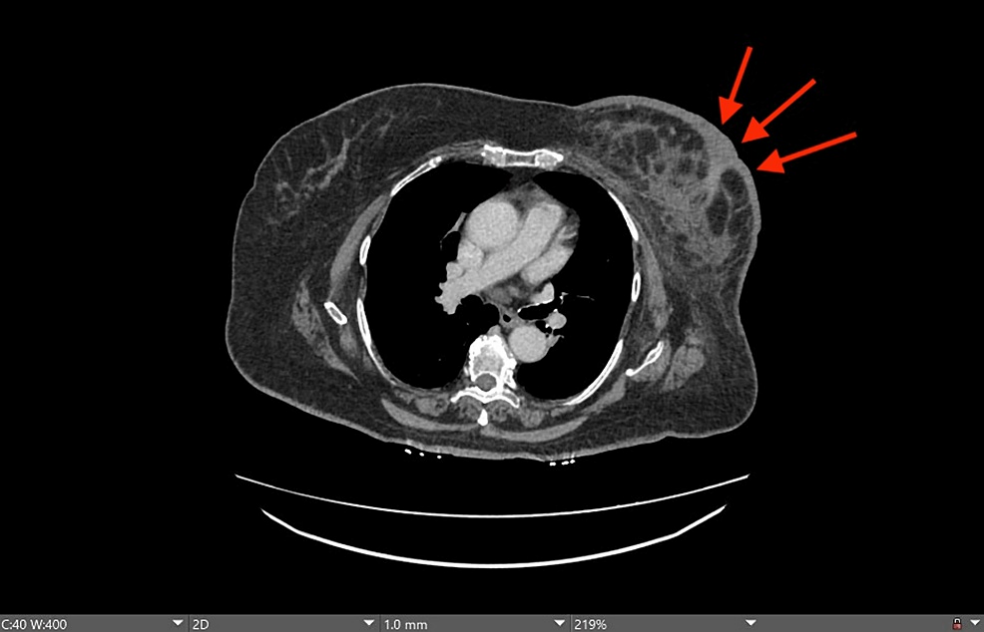
CT scan at the level of T4. Axial contrast-enhanced image demonstrating enhancement and skin thickening of the left breast. The edema and skin thickening are related to prior treatment changes based on the radiologic impression. Red arrows point to the site of the lesion.

**Figure 4:**
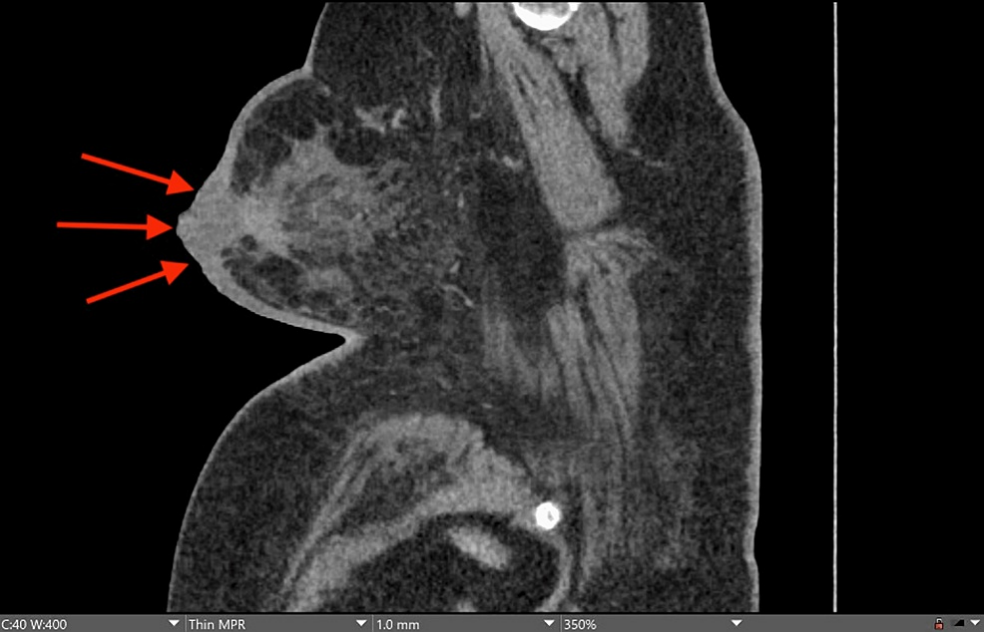
CT scan with a sagittal view. Sagittal contrast-enhanced image demonstrating enhancement and skin thickening of the left breast. The edema and skin thickening are related to prior treatment changes based on the radiologic impression. The red arrows point to the site of the lesion.

The patient was then referred for CT imaging (Figures 3-4) for further evaluation, which was interpreted as benign changes.

**Figure 5:**
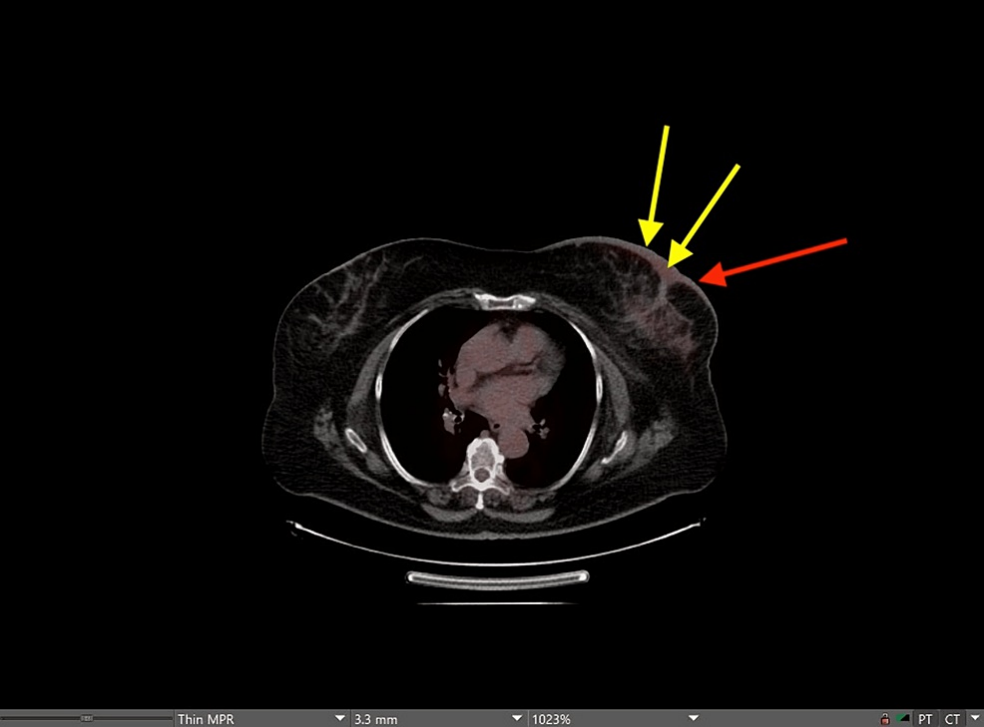
PET scan. Axial PET/CT of the left breast demonstrating increased uptake and skin thickening based on the radiologic impression. The red arrow points to the site of the lesion. Yellow arrows point to the site of increased uptake as demonstrated by the red hue on the image. PET, positron emission tomography

A mammogram repeated six months later demonstrated diffuse left breast thickening in the upper outer quadrant. Positron emission tomography-computed tomography (PET-CT) was ordered, which did not detect metastatic disease (Figure 5).

The report noted overlying skin thickening with a mild degree of metabolic activity “compatible with post-therapeutic changes.” Two months later, a punch biopsy diagnosed angiosarcoma of the breast with invasive ductal carcinoma with a tumor size of at least 9 mm grade 3. CK7 and GATA3 positive. Negative for chromogranin and synaptophysin. ER-positive (97% of nuclei), PR positive (95% of nuclei), HER2 equivocal 2+, and HER2 FISH negative. The patient was referred to surgery which suggested intervention with a 5 cm margin that would need reconstruction to close. The patient declined surgical intervention but underwent radiation therapy that she completed three months after diagnosis. The patient is currently being monitored with imaging every three months.

## Discussion

Adjuvant radiation therapy for breast carcinoma remains a cornerstone of treatment due to its efficacy in decreasing the risk of recurrence in the ipsilateral breast [7]. For patients with invasive ductal carcinoma, many women prefer to undergo a lumpectomy followed by radiation therapy and life-long hormonal therapy instead of opting for a mastectomy alone to preserve the remaining non-diseased breast [8]. However, the use of radiation can have serious consequences such as the formation of double-stranded breaks and reactive oxygen species that can permanently alter non-diseased cell DNA, potentially leading to the development of new cancer in a healthy site [9]. According to a recent study, the likelihood of developing a solid tumor showed a linear increase in relation to radiation fractions ranging from 0.15 to 1.5 Gy, which is comparable to the dose administered in the treatment of the patient presented above. [10].

The RAS, although rare, has an average five-year overall survival rate of around 63.5% [11]. Multimodal therapy, including surgery and radiation, is the primary treatment option for RAS, particularly in cases with multifocal disease. One study found that 68% of cases were managed through surgery alone, while 17% required both surgery and reirradiation [12]. This same study also revealed that surgery with reirradiation resulted in a better five-year local recurrence-free interval compared to surgery alone. For these patients, radical resection has been independently linked to reduced recurrence rates and increased five-year disease-specific survival [13]. In some cases, primary or palliative treatment may be administered without surgery, with the aim of preserving breast tissue and avoiding further surgery-related complications. As in the present case, this approach is not uncommon with 9% opting to do so [12].

The interval between radiation therapy and the development of angiosarcoma can vary widely, but the median interval is generally reported to be around 5-10 years. Several studies have reported on the timing of radiation-associated breast angiosarcoma, with varying results. For example, one study of 95 cases found a median interval of seven years between radiation therapy and the diagnosis of angiosarcoma (range 1.4-26 years), while another study of 79 cases found a median interval of seven years (range 3-19 years) [14-15]. In the above case, the patient presented with angiosarcoma five years after radiation treatment, slightly below the median time frame. Although she underwent routine imaging post-treatment with ultrasound and mammography, initial imaging could not detect any metastatic changes compared to prior imaging. It was not until the patient physically noticed the lesion that a PET scan was performed, which came back negative for malignancy and demonstrated findings consistent with post-therapeutic healing. However, post-breast cancer treatment screening guidelines remain ambiguous with some articles recommending annual diagnostic mammographic surveillance for the first three years with little evidence of the role of MRI in patients without a high risk of recurrence [16-17]. Unfortunately, mammograms and ultrasound alone have demonstrated low sensitivity in detecting RAS with one study reporting sensitivities of 43% and 50% respectively [18]. In contrast, MRI and CT are much more sensitive with 92% and 84%. Interestingly, incisional biopsies demonstrated a sensitivity of 93% in detecting RAS.

It is unclear whether the initial radiologic impressions for our patients were marred by the knowledge of potential post-therapeutic changes or if there was no other indication of malignancy. Based on the repeat imagining and the consequent negative findings, it could be the case that the diagnosis of angiosarcoma is challenging to diagnose based on breast imaging alone, and a biopsy should have been taken at the initial concern of malignancy. Unfortunately, the concern at the time of the biopsy was damage to healing skin. Due to its rarity, angiosarcoma may not have been included in the differential; if it was, MRI and biopsy should have been performed at the time, regardless of concern for healing.

## Conclusions

Radiation-associated breast angiosarcoma is a malignant consequence of breast radiation therapy that must be ruled out during follow-up of prior breast cancer treatment. Regarding differentiating between normal post-therapeutic breast changes and malignancy, a biopsy should not be postponed as imaging alone does not appear to be sensitive for diagnosis unless comparisons are made from prior studies. If imaging is to be performed, scepticism should be warranted in impressions indicating post-therapeutic changes as these can often be confused with metastatic alterations marred by prior knowledge of radiation to the area. Similarly, while ultrasound and mammography are helpful in detecting progression compared to prior imaging, these alone have a significant false-negative rates in detecting RAS. Patient reports of shooting pain and unusual appearance of the irradiated breast 4-10 years post-therapy should include angiosarcoma in the differential.
